# Challenges and Advances in the Bioproduction of L-Cysteine

**DOI:** 10.3390/molecules29020486

**Published:** 2024-01-18

**Authors:** Daniel Alejandro Caballero Cerbon, Leon Gebhard, Ruveyda Dokuyucu, Theresa Ertl, Sophia Härtl, Ayesha Mazhar, Dirk Weuster-Botz

**Affiliations:** 1 Chair of Biochemical Engineering, TUM School of Engineering and Design, Technical University of Munich, Boltzmannstraße 15, D-85748 Garching, Germany; daniel.caballero@tum.de; 2 TUM School of Engineering and Design, Technical University of Munich, Boltzmannstraße 15, D-85748 Garching, Germany; 3 TUM Campus Straubing for Biotechnology and Sustainability, Technical University of Munich, Petersgasse 5, D-94315 Straubing, Germany; ruveyda.dokuyucu@tum.de (R.D.); theresa.maria.ertl@tum.de (T.E.); ge73xob@mytum.de (S.H.);

**Keywords:** L-cysteine, biosynthesis, metabolic engineering, fermentation, fed-batch process, downstream processing

## Abstract

L-cysteine is a proteogenic amino acid with many applications in the pharmaceutical, food, animal feed, and cosmetic industries. Due to safety and environmental issues in extracting L-cysteine from animal hair and feathers, the fermentative production of L-cysteine offers an attractive alternative using renewable feedstocks. Strategies to improve microbial production hosts like *Pantoea ananatis*, *Corynebacterium glutamicum*, *Pseudomonas* sp., and *Escherichia coli* are summarized. Concerning the metabolic engineering strategies, the overexpression of feedback inhibition-insensitive L-serine O-acetyltransferase and weakening the degradation of L-cysteine through the removal of L-cysteine desulfhydrases are crucial adjustments. The overexpression of L-cysteine exporters is vital to overcome the toxicity caused by intracellular accumulating L-cysteine. In addition, we compiled the process engineering aspects for the bioproduction of L-cysteine. Utilizing the energy-efficient sulfur assimilation pathway via thiosulfate, fermenting cheap carbon sources, designing scalable, fed-batch processes with individual feedings of carbon and sulfur sources, and implementing efficient purification techniques are essential for the fermentative production of L-cysteine on an industrial scale.

## 1. Introduction

**Figure 1:**
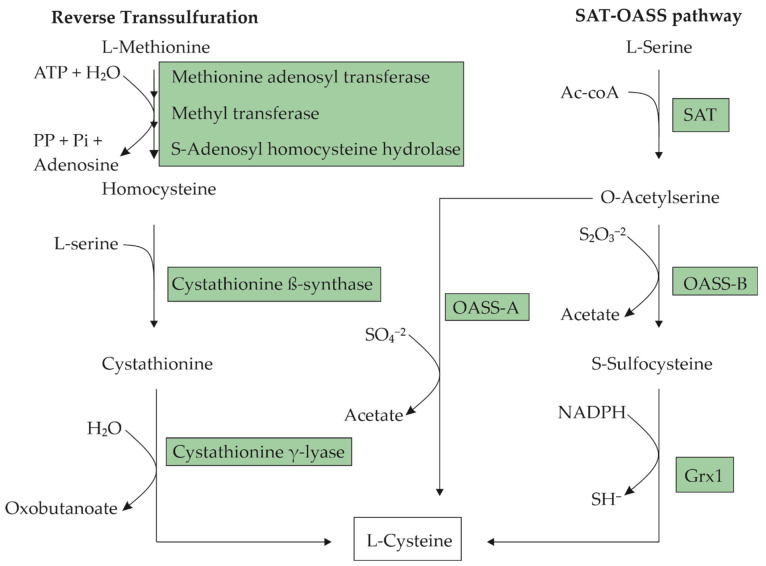
Metabolic pathways for L-cysteine biosynthesis in microorganisms. In the L-serine O-acetyltransferase-O-acetyl-L-serine (SAT-OASS) pathway, there are two branches for the transformation of O-acetyl-L-serine (OAS) into L-cysteine, depending on the sulfur source available to the organism. These biotransformations are catalyzed by two different OASSs: OASS-A in the case of sulfate as the S-source and OASS-B for thiosulfate. The transformation through OASS-B requires a subsequent reduction of S-sulfocysteine to L-cysteine catalyzed by glutathioredoxin (Grx1).

L-cysteine is a nucleophilic amino acid with a sulfhydryl group, which enables the formation of disulfide bridges. It can be produced by most eukaryotic and prokaryotic organisms. This amino acid is semi-essential in humans, since it can be synthesized endogenously or directly absorbed from an L-cysteine-rich diet. In cells, cysteine can be produced via the reverse transsulfuration pathway from L-methionine [1] or from L-serine through acetylation and subsequent sulfhydration [2]. Figure 1 presents a step-by-step diagram for these pathways. The cells tightly control the intracellular L-cysteine concentration because already-low concentrations can be cytotoxic [3,4]. Intracellular L-cysteine plays a vital role as a precursor for protein synthesis, and for the production of glutathione, hydrogen sulfide, and taurine [5]. In humans and animals, L-cysteine is primarily found in the hair, nails, and skin, especially in collagen. Prokaryotic cells use L-cysteine mostly in proteins, in enzymes for the formation of iron–sulfur clusters, or as a source of sulfur for other macromolecules [6].

L-cysteine is produced for applications in the pharmaceutical, food, animal feed, and cosmetics industries, with an estimated yearly production of roughly 3000 tons [7,8]. In the pharmaceutical and food industries, L-cysteine is a precursor for the production of drugs, food supplements, and nutraceuticals [9]. In animal feed, L-cysteine is used as a sulfur supplement for wool production [10]. In the cosmetic industry, L-cysteine is applied as a substitute for thioglycolic acid in hair applications for breaking the disulfide bridges of keratin to achieve permanent hair waving [10].

Animal feathers and human hair are natural sources of L-cysteine, which is the main component of keratin. First, cystine is extracted with activated charcoal after the acidic hydrolysis of feathers and hair. After desorption from the activated charcoal matrix, the isolated and purified L-cystine is then transformed into L-cysteine via electrolytic reduction [11]. This is the most common production method applied so far, although the L-cysteine process yield is low, and unpleasant odors may occur. However, the most important disadvantage is the environmental and safety issues caused by the disposal of the organic waste, which contains hydrochloric acid [12].

**Figure 2:**
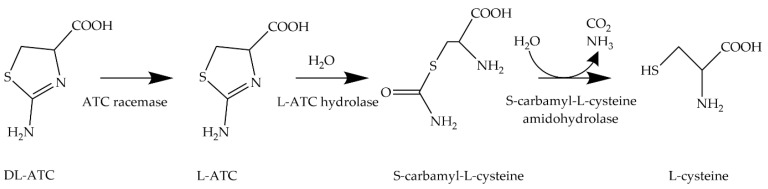
Transformation of DL-ATC to L-cysteine. In this enzymatic three-step process, DL-ATC is isomerized to L-ATC by ATC racemase. Then, L-ATC is hydrolyzed to S-carbamyl-L-cysteine (SCC) by L-ATC hydrolase. Finally, SCC is cleaved into L-cysteine, CO_2_, and ammonia by S-cabamyl-L-cysteine amidohydrolase.

An alternative approach is the biotechnological production of L-cysteine from renewable resources [13]. Since the 1980s, microorganisms of the genus *Pseudomonas* have been used for the asymmetric whole-cell biocatalysis of DL-2-amino-Δ^2^-thiazoline-4-carboxylic acid (DL-ATC) to generate L-cysteine (Figure 2).

*Pseudomonas* is a genus of gram-negative bacteria belonging to the family Pseudomonadaceae in the class of γ-proteobacteria. From the many bacteria, especially in the genus *Pseudomonas*, that have an activity to convert DL-ATC to L-cysteine, two biosynthetic pathways have been described [14,15,16]. As an intermediate, one pathway produces N-carbamoyl cysteine, and the other, S-carbamoyl cysteine [17]. However, in the *Pseudomonas* cells, L-cysteine is converted to pyruvate, hydrogen sulfide, and ammonia by L-cysteine desulfhydrase (CD). The L-cysteine desulfhydrase gene could not be knocked out to increase the production of L-cysteine during the bioconversion of DL-ATC by *Pseudomonas* sp., since the degradation of L-cysteine is required to supply the cells with carbon and nitrogen [18]. Furthermore, the difficulty of procurement of the substrate DL-ATC and the low enzymatic yields of this process limited its application as an industrial process [12].

Through the understanding of the complex regulation system of the L-cysteine synthesis in bacteria, and with the advancements in synthetic biology, the direct fermentative L-cysteine production from glucose became feasible and has been exploited by global amino acid producers to supply an increasing portion of the global L-cysteine demand [19].

In this review, the metabolic engineering strategies for enhancing the L-cysteine yield and cell-specific productivities will be compiled, e.g., improving the metabolic availability of precursors, deregulating controlling enzymes, knocking out genes coding for L-cysteine-degrading enzymes, and overexpressing transporter genes to transport L-cysteine out of the cell. The final section of this review focuses on the process engineering aspects like sulfur sources, fermentation process designs, and L-cysteine purification strategies.

## 2. Metabolic Engineering Strategies

Microorganisms use two main pathways for the biosynthesis of L-cysteine. In enteric bacteria such as *E. coli*, the precursor of L-cysteine is L-serine [20]. L-cysteine is synthesized from L-serine in two steps, which involve the substitution of the β-hydroxyl group with a thiol group (Figure 1). The first step involves the acetylation of the β-hydroxyl of L-serine, resulting in an O-acetyl-L-serine (OAS) catalyzed by L-serine O-acetyltransferase (SAT), which is encoded by the *cysE* gene. The second step involves the α,β-elimination of acetate from OAS and the addition of H_2_S to give an L-cysteine catalyzed by O-acetyl-L-serine sulfhydrylase-A (OASS-A), which is encoded by the *cysK* gene. *Corynebacterium glutamicum* and *Pantoea ananantis* synthesize L-cysteine through the same pathway as *E. coli* [12]. Other microorganisms, such as *Pseudomonas putida* [21], *Saccharomyces cerevisiae* [22], and *Lactococcus lactis* [23] synthesize L-cysteine from L-methionine through the reverse transsulfuration pathway that consists in the cleavage of a methyl group from L-methionine to generate a homocysteine catalyzed by a multienzyme complex, and the homocysteine’s subsequent transformation to cystathionine by the enzyme cystathionine beta-synthase (CBS). L-cysteine can be produced from cystathionine through the enzyme cystathionine gamma-lyase (CSE) (Figure 1).

The choice of the starting strain for metabolic engineering has an impact on the potential enhancements achieved through the modification of metabolic steps [24]. This will be shown in an example with *E. coli*: It has been established that the transcription levels of key metabolic genes are the major modulators of different glucose utilization pathways in *E. coli* [25]. Also, the genetic difference in the catabolite repression protein (CRP) among the *E. coli* K-12 strains affects sulfur metabolism [26]. During the last 20 years, four *E. coli* K-12 strains (BW25113, JM109, W3110, and MG1655, respectively) have been utilized in L-cysteine production [27]. Therefore, the selection of a suitable chassis for L-cysteine production has been deemed the foundation for the successful implementation of metabolic engineering strategies [28]. To reach a more suitable chassis cell, the L-cysteine-producing plasmid pLH03, containing P*-cysE* (coding for serine acetyltransferase) and P*-ydeD* (a cysteine exporter), was transformed into the four aforementioned *E. coli* K-12 hosts. The results of shake flask experiments (minimal medium with thiosulfate as the sulfur source) showed that the best chassis for the plasmid pLH03 was BW25113, since it produced 345.8 mg L^−1^ of L-cysteine, followed by JM109 (190.3 mg L^−1^). By contrast, neither W3110 nor MG1655 were deemed suitable base strains for this plasmid, since they each produced less than 70 mg L^−1^ product. [28]

Henceforth, the advances in the metabolic engineering of a variety of organisms will be discussed. In general, the fermentative production of L-cysteine can be enhanced by utilizing a combination of three different metabolic engineering strategies, which are as follows:

- Enhancing the biosynthesis of L-cysteine by expressing genes encoding feedback inhibition-insensitive phosphoglycerate dehydrogenase (PGDH) L-serine O-acetyltransferase (SAT);
- Weakening the degradation of L-cysteine by knocking out genes encoding L-cysteine desulfhydrase (CD);
- Enhancing and strengthening the efflux system while weakening and deleting the genes responsible for the import of L-cysteine.

Table 1 at the end of this chapter summarizes the maximal L-cysteine concentrations achieved by applying these metabolic engineering approaches, as well as the organism and scale of the process by which they were reached.

### 2.1. Biosynthesis of L-Cysteine

The acetylation of L-serine catalyzed by serine acetyltransferase (SAT) is the rate-limiting step of L-cysteine biosynthesis in many microorganisms. The activity of SAT is suppressed by L-cysteine via feedback inhibition (Figure 2). An approach to enhance the fermentative production of L-cysteine utilizes feedback inhibition-insensitive SATs. These SATs are obtained by the following means:

- Engineering SAT from *E. coli* through site-directed mutagenesis or random mutagenesis [29,30];
- Utilizing natural SAT from higher plants insensitive to feedback inhibition [31,32].

Nakamori et al. [33] obtained feedback inhibition-insensitive SATs by engineering SAT from *E. coli* through site-directed mutagenesis using PCR. In this study, the *cysE* gene coding for SAT was altered by replacing the methionine at position 256 with other amino acids. The transformed microorganisms, having the altered *cysE* gene, produced 50–300 mg L^−1^ of L-cysteine plus L-cystine after 72 h of cultivation in shake flasks containing a minimal medium at 30 °C. Furthermore, the transformation of the altered *cysE* gene in a mutant strain of *E. coli*, JM39-8, with decreased cysteine degradation abilities (10% CD activity) resulted in an increased production to a maximum of 790 mg L^−1^ of L-cysteine plus L-cystine in shake flasks under the same conditions as above. Takagi et al. [30] isolated feedback inhibition-insensitive SAT through a random mutagenesis into the *cysE* gene by using the error-prone PCR. The production test of L-cysteine and the enzymatic analysis of the mutant SAT suggested that the carboxy-terminal region of SAT is involved in the desensitization to feedback inhibition and the substantial production of L-cysteine.

Another approach to obtain feedback inhibition-insensitive SATs could be to use natural SATs from higher plants. In *Arabidopsis thaliana*, three cDNA clones encoding SAT isoforms (SAT-c, SAT-p, and SAT-m) have been isolated. Noji et al. [33] analyzed these SATs and reported that SAT-p and SAT-m were feedback inhibition-insensitive isozymes. However, the activity of SAT-c was feedback inhibited by a low concentration of L-cysteine. Takagi et al. [31] expressed two cDNAs from *A. thaliana* encoding feedback inhibition-insensitive SAT (SAT-p and SAT-m) in the *E. coli* strain JM39-8, and reported an increased production of L-cysteine (1.6–1.7 g L^−1^) when cultivated for 96 h in shaking flasks containing a minimal medium at 30 °C. Wirtz and Hell [32] isolated three new cDNAs from *Nicotiana tabacum* encoding SATs and reported that the SAT4 isoform has a lower feedback sensitivity than the previously reported SATs. The results showed that the expression of SAT4 in *E. coli* causes a 50-fold increase in the accumulation of L-cysteine in the medium.

Kai et al. [34] analyzed the crystal structure and reaction mechanism of SAT and introduced various amino acid substitutions to change the position of Asp92. As a result, they designed a mutant SAT having a high enzymatic activity and low sensitivity to inhibition by L-cysteine. For one promising mutant, the inhibitory L-cysteine concentration that halved the enzyme’s activity went from the wild type’s 0.8 µM to 410 µM, while simultaneously increasing the initial enzymatic activity by 220 µM mg^−1^ min^−1^.

In another study, a strain of *P. ananatis* that carried a mutant of the gene *cysE (cysEX)* coding for a feedback-insensitive SAT was constructed. A second strain of *P. ananatis*, additionally harboring a plasmid with the gene *cefA*, which codes for an L-cysteine efflux pump, was generated. A strain of *P. ananatis* carrying the gene *cefA* was able to produce 900 mg L^−1^ of L-cysteine in agitated test tubes, which is roughly four times the amount produced by the *P. ananatis* strain carrying only the feedback-insensitive SAT [6].

*C. glutamicum* is a nonpathogenic and high G+C gram-positive bacterium industrially used for decades for the fermentative production of amino acids such as L-glutamic acid and L-lysine [35,36]. Joo et al. [37] investigated the effect of the overexpression of genes encoding SAT, OASS, and a transcriptional regulator in *C. glutamicum*. The results showed that L-cysteine production was enhanced by approximately three-fold, with the recombinant strain reaching a maximal L-cysteine concentration of approximately 60 mg L^−1^ in shake flasks at 30 °C after 15 h of cultivation. Wei et al. [7] further engineered *C. glutamicum* for L-cysteine production by enhancing the L-serine biosynthetic pathway, expressing the gene encoding feedback inhibition-insensitive SAT, deleting genes encoding CD, and increasing the L-cysteine export. Their engineered strain, cultivated for 48 h at 30 °C in shake flasks, produced 947.9 ± 46.5 mg L^−1^ of L-cysteine, with a yield of 27.27 mg g^−1^ of glucose and a volumetric productivity of 26.33 mg L^−1^ h^−1^. Kondoh and Hirasawa [38] achieved approximately 200 mg L^−1^ of L-cysteine production with *C. glutamicum* by overexpressing the mutant *cysE* and *serA* genes to enhance the biosynthesis, disrupting *aecD* gene coding for CD to prevent degradation and disrupting the NCgl2463 gene coding for a membrane transporter to prevent L-cystine import.

One of the key enzymes in the L-serine biosynthesis pathway, 3-phosphoglycerate dehydrogenase (PGDH), coded by the gene *serA*, is feedback inhibited by L-serine [7,13]. The authors of [39] converted the 344th and 346th amino acids of *E. coli*’s PGDH to alanine, which created a feedback-insensitive *serA*. Liu et al. [13] overexpressed the feedback-resistant *serA* as well as wild-type variants of the two subsequent enzymes of the pathway, phosphoserine transferase (*serB*) and phosphoserine phosphate-lyase (*serC*), to produce L-serine. The *E. coli* strain accumulated 11.7 g L^−1^ of L-serine after 48 h in shake flask experiments with glucose as the carbon source, which was the highest concentration so far.

From these results, it was concluded that the overexpression of *serA* along with *serC* and *serB* in the L-serine synthesis pathway was effective in increasing the production of L-cysteine, which resulted in L-cysteine concentrations in shake flask experiments of about 492.0 mg L^−1^, with a yield of about 47 mg of L-cysteine g^−1^ glucose in 48 h [13].

### 2.2. Weakening the Degradation of L-Cysteine

It is well known that disrupting the degradation pathway and enhancing metabolic fluxes for the desired amino acids are effective approaches to improve their production [19,39,40,41]. The degradation of L-cysteine to pyruvate, ammonia, and sulfide is mainly catalyzed by L-cysteine desulfhydrase (CD). Wada et al. [42] purified and characterized CD from *C. glutamicum*. After comparing the partial amino acid sequence, it was identified that the enzyme CD is a C-S lyase with α,β-elimination activity, and it is encoded by the *aecD* gene [43]. They showed that the *aecD* gene product is involved in L-cysteine degradation, and the disruption of the *aecD* gene resulted in an increased production of L-cysteine (approximately 290 mg L^−1^ in shake flasks).

Awano et al. [44] investigated the enzyme having CD activity in *E. coli* using native-PAGE and CD activity staining. An analysis with gene-disrupted mutants showed that the tryptophanase (TNase) encoded by *tnaA* and cystathionine β-lyase (CBL) encoded by *metC* catalyze the degradation of L-cysteine in *E. coli*. By using a plasmid gene library, Awano et al. [45] identified three additional proteins as O-acetylserine sulfhydrylase-A (OASS-A), O-acetylserine sulfhydrylase-B (OASS-B), and the bifunctional ß-cystathionase (MalY), all having CD activity in *E. coli*. The disruption of these CD genes in *E. coli* resulted in the increased production of L-cysteine and L-cystine after 72 h by factors of 1.8 to 2.3 in shake flasks. The five above-mentioned proteins are pyridoxal 5′-phosphate (PLP)-dependent enzymes, and cysteine desulfhydrase activity might just be a side reaction.

The transcriptional activator CysB, belonging to the LysR family, induces the transcription of genes in the sulfate pathway [46]. Kawano et al. [46] found a most plausible sequence for the CysB-binding motif upstream of the *yciW* gene. YciW encodes a soluble protein of 401 amino acids, although its function and structural information are still unknown. The CysB-binding motif is located at the −35 region of the predicted promotor, which is similar to that of *cysJ* coding for a sulfite reductase. The *yciW*-disrupted strain exhibited a much higher sensitivity to L-cysteine than the native strain. This indicates that *yciW* confers tolerance to cysteine in *E. coli* cells, and the authors suggest that *yciW* may convert L-cysteine to L-methionine or glutathione. Based on the deduced amino acid sequence, *yciW* is predicted to encode an oxidoreductase-like protein. YciW is also involved in the degradation of intracellular L-cysteine, leading to its detoxification. However, further investigation is needed at the protein level [46].

L-cysteine desulfidases are alternative L-cysteine-degrading enzymes that are less well characterized as compared to L-cysteine desulfhydrases. The activity of this enzyme was first observed in *Methanocaldococcus jannaschii* [47]. Nonaka and Takumi [48] identified the *yhaM* gene, which encodes cysteine desulfidase and decomposes L-cysteine into hydrogen sulfide, pyruvate, and ammonium in *E. coli*. They reported that *yhaM* is L-cysteine inducible; therefore, it could serve as the main target for metabolic engineering to enhance L-cysteine production. Loddeke et al. [49] reported that L-cysteine desulfidase (CyuA) is the major enzyme that degrades L-cysteine during anaerobic growth in both *E. coli* and *Salmonella enterica*. In their research, *ΔcyuA* mutants of both species grown with L-cysteine anaerobically presented a reduced growth capacity and a 57% reduction in their sulfur production. The authors postulated that the presence of *cyuA* is a vestige predating the separation of bacteria and archaea and has three possible functions in bacteria: the use of L-cysteine as a carbon source, the detoxification of L-cysteine at growth-inhibiting concentrations, and the modulation of intracellular L-cysteine levels at nontoxic concentrations.

Takumi et al. [6] identified a major L-cysteine desulfhydrase gene (*ccdA*) involved in L-cysteine degradation in *Pantoea ananatis*. In their study, the *E. coli* wild-type strain MG1655 was transformed with a multicopy genomic library prepared from *P. ananatis* genomic DNA. From the investigated genes, the genes *ccdA*, which presents a cysteine degradation activity, and *cefA* and *cefB*, which are putative efflux pumps, were found to have a significant impact on the L-cysteine production from glucose and thiosulfate in experiments performed with *E. coli* in agitated test tubes. The overexpression of *ccdA* in *P. ananatis* led to a decrease of 58% in the maximal achieved L-cysteine concentration in test tubes. Conversely, the deletion of *ccdA* resulted in an increase in the achieved L-cysteine concentration in test tubes from 202 mg L^−1^ with the wild type to 516 mg L^−1^.

For the accumulation of L-cysteine in *C. glutamicum*, the deletion of the enzyme CD, encoded by *aecD*, and the overexpression of the native CysE, encoded by *cysE*, were performed [7]. In contrast to the wild-type strain that produced 7.1 ± 1.5 mg L^−1^ of L-cysteine on a shake flask scale, the CD-deleted *C. glutamicum* strain accumulated 10.6 ± 2.3 mg L^−1^ of L-cysteine, thus showing no significant improvement. After the overexpression of a *CysE* mutant from *E. coli*, coding for a feedback-insensitive serine acetyltransferase in the CD deletion strain, an L-cysteine concentration of 346.1 ± 33.1 mg L^−1^ was measured. This result indicates that the combination of the overexpression of a feedback-insensitive CysE and deletion of CD is necessary to enhance L-cysteine production with *C. glutamicum* [7,50].

### 2.3. Regulation of L-Cysteine Transport

The bacterial production of metabolites depends on the equilibrium between secretion and reconsumption. High concentrations of L-cysteine have been reported to be toxic and inhibitory for microbial cells [3]. In *E. coli*, concentrations of 1 mM L-cysteine were enough to cause growth limitations [46]. Therefore, microorganisms have an exporter for L-cysteine within the L-cysteine regulon in order to avoid product-associated cytotoxicity.

Daßler et al. [51] identified the transporter protein YdeD in *E. coli*, which is reported to be one of the main facilitators involved in the export of L-cysteine. The corresponding gene encoding for the transporter protein involves an open reading frame coding for 299 amino acids. The substrate range of the YdeD protein is wide; therefore, it secretes other metabolites along with L-cysteine. In addition, YdeD acts as a regulator by exporting O-acetylserine (OAS), therefore lowering the intracellular NAS concentration. This stops the L-cysteine regulon activation [52]. The combination of YdeD with the strong promoter from the gene cassette coding for glyceraldehyde-phosphate dehydrogenase GAPDH in a plasmid generated a higher yield of L-cysteine with *E. coli*, proving the transporter’s vital role in the accumulation of the metabolite [13].

CydDC is an ATP-binding transporter reported to be involved in L-cysteine export from the cytoplasm to the periplasm in *E. coli* [53]. In a study, CydDC was compared to the gene product of *ydeD* and was found to more effectively remove high L-cysteine concentrations in intracellular compartments than *ydeD*. In shake flask experiments at 37 °C, *E. coli* mutants with deletions of *cydDC* accumulated 1.6 times higher L-cysteine concentrations than the wild type, while mutants with *ydeD* deletions accumulated 1.3 times higher L-cysteine concentrations. A double-deletion *E. coli* mutant of both *cydDC* and *ydeD* accumulated 2.2 times more L-cysteine than the wild type. [53]

*yfiK* was discovered to be a gene that augmented L-cysteine production when it was overexpressed in an *E. coli* production strain [52]. The gene product is an integral membrane protein with about six predicted transmembrane helices and belongs to the resistance to homoserine/threonine (RhtB) family of export proteins. YfiK overproduction from a plasmid leads to the drastic and parallel secretion of O-acetyl serine and L-cysteine into the medium, but only when the *E. coli* strain possesses an L-serine transacetylase that is feedback insensitive to L-cysteine. Externally provided, OAS obviated this requirement for L-cysteine secretion, both in the *yfiK-*carrying transformant and in the wild type. A Δ*yfiK* mutant did not show any phenotype, and this mutant exported OAS and L-cysteine when transformed with a plasmid carrying *ydeD*, a previously characterized, alternate OAS/L-cysteine exporter. Since a *ydeD–yfiK* double mutant showed the same pattern, it appears that YfiK and YdeD act independently. The necessity for the cell to regulate the size of the internal pool of OAS via the synthesis of exporter proteins could be connected to the fact that this compound inhibits growth when supplied externally. The overexpression of either *ydeD* or *yfiK* leads to alleviation of this inhibition parallel by an increased resistance to azaserine, which is an analog of OAS [52].

In another study, different drug transporter genes were tested, and *Bcr* (a multidrug transporter) was found to be involved in L-cysteine export with an increased selectivity compared to other amino acids [54].

Besides these cytoplasmic membrane transporters, a novel gene called *tolC*, which encodes the outer membrane channel TolC, was reported to be involved in L-cysteine export from the periplasm in the medium [55].

The genes *cefA* and *cefB* were identified as cysteine efflux pumps. In a first experiment, the overexpression of *cefA* and *cefB* in *P. ananatis* resulted in L-cysteine resistance when 200 µM of L-cysteine was added to the growth medium. When the *P. ananatis* strains overexpressing either *cefA* or *cefB* were grown in aerated test tubes in the presence of glucose and thiosulfate, the maximum final L-cysteine concentrations were increased by 0.2 g L^−1^ and 1.9 g L^−1^, to maximal concentrations of 0.4 g L^−1^ and 2.3 g L^−1^, respectively, compared to *P. ananatis,* without the overexpression of these efflux pump genes [6].

The genes NCgl0580 and NCgl2566 were identified as potential L-cysteine exporters in *C. glutamicum*, and it was found that their overexpression in a *C. glutamicum* strain increased the L-cysteine formation from glucose and thiosulfate. This strain achieved a maximal L-cysteine concentration of 280 mg L^−1^ in shake flasks, which is a 1.4-fold improvement compared with the original *C. glutamicum* strain [56].

Wei et al. [7] evaluated the effectivity of three heterologous proteins responsible for L-cysteine transport in *C. glutamicum*. The transporters YdeD and Bcr from *E. coli* and CefA from *P. ananatis* were individually overexpressed. While all three strains overexpressing L-cysteine transporters have shown improvements in L-cysteine production, the best results were observed with Bcr, followed by YdeD and CefA. The strain with Bcr achieved an 88.9% increase in the L-cysteine concentration in shake flasks (634.4 mg L^−1^) compared to the strain without additional transporters. The results indicated that the overexpression of these proteins enhanced the secretion of L-cysteine, preventing its accumulation in the cytoplasm of *C. glutamicum* cells and, therefore, improving cell growth. The levels of L-cysteine obtained from the engineered strains were still approximately five times lower in comparison with the results of [54], whose authors performed the same alteration on the *Bcr* and *YdeD* genes in *E. coli* cells. This difference is believed to be caused by the different cell membrane structures of the two bacteria, with *C. glutamicum* being gram positive and *E. coli* gram negative [7].

Among L-cysteine transport proteins, there are few that are solely responsible for the import of L-cysteine back into the cytoplasm. This is disadvantageous for the industrial biosynthesis of L-cysteine, since it doesn’t allow for the accumulation of the product and causes backflow. In a recent study, the *yeaN* gene product, a known 2-nitroimidazole exporter, was found to take part in L-cysteine import in *E. coli* strains [57]. The deletion of the *yeaN* gene in an L-cysteine producer strain increased the overall L-cysteine production by 50% in shake flasks.

A recent study by Du et al. [58] has shown how to regulate the L-cysteine transport system in *C. glutamicum* effectively to establish intracellular homeostasis, avoiding toxicity. This was achieved by the deletion of *NCgl2463*, an L-cysteine importer gene [37], the upregulation of the export gene *eamA*, and the enhancement of the NADPH pool through the overexpression of glucose-6-phosphate dehydrogenase. These genetic modifications resulted in a producer strain that, in a 5 L stirred-tank reactor, was able to accumulate 5.92 g L^−1^ of L-cysteine with a sulfur conversion rate of nearly 75%.

**Table 1:** Summary of metabolic engineering approaches increasing the fermentative L-cysteine production. The studies presented in this table are ordered by the kind of approach each investigation followed, as well as the date of the publication. Only the highest L-cysteine concentration of each publication is listed, together with the molecular biology step applied in order to reach that specific concentration.

Source	Organism	Method	Scale	L-Cysteine Concentration
Enhancing biosynthesis of L-cysteine				
[28]	*E. coli* JM39-8	Feedback inhibition-insensitive SAT from site-directed mutagenesis	Shake flasks, 72 h	0.79 g L^−1^
[30]	*E. coli* JM39-8	Insensitive SAT from *Arabidopsis thaliana*	Shake flasks, 96 h	1.66 g L^−1^
[31]	*E. coli* MW1	Insensitive SAT from *Nicotiana tabacum*	Shake flasks, 72 h	0.30 g L^−1^
[6]	*P. ananatis*	Insensitive SAT with L-cysteine efflux pump	Test tubes, 24 h	0.90 g L^−1^
[36]	*C. glutamicum*	Overexpression of native SAT, OAS, and transcription regulator	Shake flasks, 15 h	0.06 g L^−1^
[7]	*C. glutamicum*	Insensitive SAT, deletion of CD, and increased transport	Shake flasks, 48 h	0.95 g L^−1^
[37]	*C. glutamicum*	Overexpression of SAT and PGDH with disruption of CD and L-cysteine import	Shake flasks, 12 h	0.20 g L^−1^
[13]	*E. coli*	Overexpression of PGDH, PSERT, and PSL	Shake flasks, 48 h	0.49 g L^−1^
Weakening degradation of L-cysteine				
[41]	*C. glutamicum*	Disruption of *aecD* gene (CD)	Shake flasks, 72 h	0.29 g L^−1^
[44]	*E. coli*	Disruption of TNase, CBL, OASS-A, OASS-B, and MalY	Shake flasks, 72 h	1.36 g L^−1^ (per OD_562_)
[46]	*E. coli*	Disruption of the *yciW* gene	Shake flasks, 72 h	0.31 g L^−1^ (per OD_562_)
[6]	*P. ananatis*	Deletion of CD *ccdA*	Test tubes, 28 h	0.52 g L^−1^
[7]	*C. glutamicum*	Disruption of *aecD* (CD) and overexpression of insensitive SAT	Shake flasks, 48 h	0.35 g L^−1^
Enhancing efflux of L-cysteine				
[51]	*E. coli*	Overexpression of exporter YdeD	Shake flasks, 48 h	0.07 g L^−1^
[52]	*E. coli*	Overexpression of exporter YfiK	Shake flasks, 20 h	0.15 g L^−1^
[54]	*E. coli*	Overexpression of exporter bcr and deletion of TNase	Shake flasks, 48 h	0.50 g L^−1^
[55]	*E. coli*	Overexpression of exporters YdeD and TolC	Shake flasks, 24 h	0.12 g L^−1^
[6]	*P. ananatis*	Overexpression of transporter *cefB*	Test tubes, 28 h	2.30 g L^−1^
[56]	*C. glutamicum*	Overexpression of transport genes NCgl0580 and NCgl2566	Shake flasks, 20 h	0.28 g L^−1^
[7]	*C. glutamicum*	Overexpression of exporter bcr	Shake flasks, 48 h	0.63 g L^−1^
[57]	*E. coli*	Deletion of importer *yeaN*	Shake flasks, 20 h	1.20 g L^−1^
[58]	*C. glutamicum*	Deletion of importer *NCgl2463*, overexpression of exporter *eamA* and G6PDH	5 L stirred tank, 72 h	5.92 g L^−1^

OD_562_ is the optical density of the fermentation broth measured at 562 nm.

### 2.4. Limitations of the Metabolic Engineering Approaches

Liu et al. [13] developed an integrated approach, which used many of the previously presented metabolic engineering strategies in tandem. They overexpressed PSERT, PSL, and a feedback-insensitive version of PGDH, disrupted genes for the degradation of L-serine and L-cysteine, and overexpressed the L-cysteine exporter YdeD. Even though this strategy showed a cumulative improvement in the L-cysteine concentration, the final yield of L-cysteine on glucose was about 4.7% (*w*/*w*). This means that most of the fed substrate was not directly utilized for the L-cysteine production. Nonetheless, the authors indicate that this is the highest yield on glucose found in the literature at the time of its publication.

This exemplifies one of the major challenges of the metabolic engineering approaches. Most of the methods described above are applied directly on the L-cysteine synthesis pathway, which has been extensively characterized so that the bottlenecks are extensively identified. However, most metabolic pathways are thoroughly interconnected with regulation interactions that have not been yet characterized [59]. These interactions must then first be mapped out in order to identify further bottlenecks that can be tackled through rational metabolic engineering methods [59].

Furthermore, this interconnectivity of metabolic pathways may also result in unintended consequences from seemingly straightforward molecular biology interventions, like the lack of growth observed in *Pseudomonas* sp. by Huai et al. [18] when an enzyme responsible for L-cysteine degradation was deleted, or the increase in the exogenous L-cysteine concentration leading to lower levels of c-type cytochromes and increased penicillin resistance [53].

## 3. Process Engineering Aspects

Metabolic engineering of the microorganisms used for L-cysteine biosynthesis has been applied to enable L-cysteine production by fermentation. However, the economic L-cysteine production by fermentation must also take into account the process engineering viewpoint. Emphasizing energy-efficient sulfur pathways [7,60], selecting economically viable carbon sources [61], designing scalable fed-batch processes with individual feedings of carbon and sulfur sources [59], and implementing efficient purification techniques [62] are important for the industrial-scale production of L-cysteine.

### 3.1. Sulfur Assimilation Pathways in Bacteria

*E. coli* has two sulfur assimilation pathways for L-cysteine biosynthesis (Figure 2). One is the sulfate (SO_4_^2−^) pathway, which consumes two molecules of ATP and four molecules of NADPH as a reducing power to produce L-cysteine from sulfate. The other is the thiosulfate (S_2_O_3_^2−^) pathway, which spends only one molecule of NADPH from thiosulfate [63]. Thiosulfate is receiving attention as a sulfur source due to its advantageous effects on bacterial growth and L-cysteine production in comparison to sulfate [64].

Both sulfur assimilation pathways utilize OAS as a carbon skeleton to which they may incorporate sulfur. In the thiosulfate pathway, O-acetyl-L-serine sulfhydrylase B (CysM) catalyzes the conversion of OAS and thiosulfate into S-sulfocysteine, which is reduced into L-cysteine and sulfite by glutaredoxin (Grx1), and the glutaredoxin-like protein NrdH [63,65]. In comparison, sulfate is first reduced to sulfide in a four-step reaction catalyzed in subsequent order by sulfate adenylyltransferase (CysDN), adenylylsulfate kinase (CysC), 3′-phospho-adenylylsulfate reductase (CysH), and sulfite reductase (CysIJ). The subsequent incorporation of sulfide into OAS is enabled by O-acetyl-L-serine sulfhydrylase A (CysK) [60].

There is strong evidence suggesting that *E. coli* may possess an alternative pathway for assimilating thiosulfate, independent of the conventional pathway through the CysM enzyme [64]. This evidence comes from an experiment where ∆CysM *E. coli* cells were grown using thiosulfate as the only sulfur source. The ∆CysM cells showed a slower growth compared to the wild-type cells. However, when L-cystine was added to the growth media, the impaired growth was restored, indicating a deficiency in L-cysteine. The growth rate in the growth media without L-cystine was then increased by overexpressing thiosulfate sulfurtransferase (GlpE). Based on these findings, researchers postulated that the CysM-independent pathway involves the direct breakdown of thiosulfate into sulfite by GlpE, followed by assimilation through the sulfate pathway [64]. The proposed sulfur assimilation pathway, as summarized in Table 2, is more energy efficient than the sulfate pathway, but less efficient than the traditional thiosulfate-based sulfur assimilation pathway. The energy efficiency of the sulfur assimilation pathway influences the fermentative production of L-cysteine significantly. This has been shown as increasing the levels of GlpE and CysK, which are responsible for the CysM-independent pathway, enhances L-cysteine overproduction only in wild-type *E. coli* strains, while the same modifications worsen L-cysteine overproduction in strains with elevated expressions of CysM and nrdH, responsible for the traditional, energy-efficient thiosulfate γ pathway [66].

### 3.2. Glycerol as Carbon Source

Although glucose remains the predominant carbon source for L-cysteine biosynthesis, glycerol has gained increasing attention as an alternative carbon source due to its abundant availability as a by-product in biodiesel production [66]. Utilizing glycerol as a carbon source is crucial for reducing the costs of bioproduction and improving resource utilization. Moreover, the metabolic pathway from glycerol to L-cysteine is shorter and exhibits better carbon economy compared to glucose [67].

Significant progress has been made towards the bioproduction of L-cysteine from glycerol, as its essential precursor, L-serine, has already been produced effectively using glycerol [67]. The highest recorded concentration of L-cysteine from glycerol achieved so far is 313.4 mg L^−1^ with *E. coli* in shake flasks [67]. First, L-serine production was enhanced by making use of an L-serine biosensor based on the transcriptional regulator NCgl0581 for coupling L-serine biosynthesis to the growth rate of *E. coli* and using adaptive laboratory evolution (ALE). Following this, the L-cysteine degradation pathway (encoded by *tnaA*) was eliminated, while serine acetyltransferase (encoded by *cysE*) and the L-cysteine exporter (encoded by *ydeD*) were overexpressed [67].

### 3.3. Fed-Batch Processes in Stirred-Tank Bioreactors

Up to this point, most research presented had been carried out as simple batch processes in shake flasks, which allow for easier preparation and higher throughput than the scalable stirred-tank bioreactor. Investigations in fed-batch operated and fully controlled stirred-tank bioreactors using the observations provided by the studies in shake flasks and optimizing the reaction conditions may lead to an L-cysteine production scenario that is more in line with the expectations of the industry.

Liu et al. [13] utilized a rational approach which combined previously discussed metabolic engineering approaches to enhance the L-cysteine production of the *E. coli* strain JM109, including the overexpression of the enzymes in the L-serine biosynthetic pathway, feedback-insensitive phosphoglycerate dehydrogenase and serine acetyltransferase, the ydeD exporter, and the deletion of L-cysteine and serine deaminases. These modifications lead to a total L-cysteine production in shake flasks of 600 mg L^−1^. They then cultivated this modified strain in a 5 L stirred-tank reactor in a fed-batch operation, where a glucose solution was fed constantly into the reactor, but thiosulfate (the sulfur source) was only supplied at the beginning of the process. This led to an increase in the maximal achieved L-cysteine concentration to 5.1 g L^−1^.

**Figure 3:**
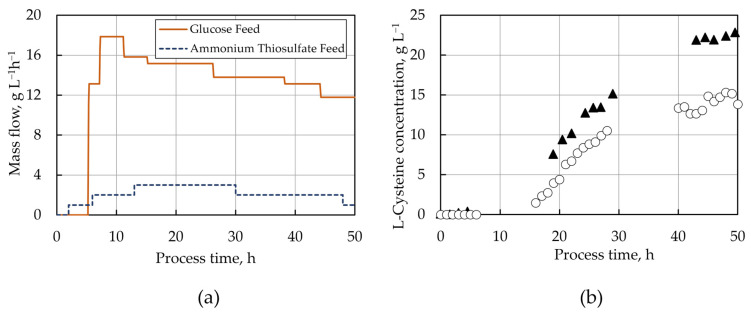
(**a**) Substrate feeding profiles followed by [59] in the fed-batch L-cysteine production process. The thiosulfate feeding started two hours after the process was inoculated in order to avoid unnecessary oxidative stress on the cells in the initial lag phase. The glucose feeding started after five hours, once the initially added glucose was completely consumed. (**b**) L-cysteine concentration profiles from the fermentation processes carried out with the *E. coli* strain W3110, with the L-cysteine production plasmid and without the overexpression of OASS-A (circles), and the same strain and plasmid but additional overexpression of OASS-A (triangles).

Caballero et al. [59] transferred the shake flask experiments of Daßler et al. [51] with *E. coli* W3110, carrying L-cysteine production plasmids to a stirred-tank bioreactor on a 15 L scale using continuous but independent glucose and ammonium thiosulfate feedings (presented in Figure 3a), thereby increasing the maximal achieved L-cysteine concentration from 72 mg L^−1^ in shake flasks to 16 g L^−1^ in the fed-batch process. An in vivo metabolic control analysis (MCA) was applied after rapid media transition [68] and short-term perturbation experiments [69,70] for the identification of bottlenecks in the L-cysteine synthesis pathway of producer cells withdrawn from this fed-batch process in the production phase. Synthetic biology efforts to increase the expression of OASS-A based on the results of the in vivo MCA led to an increase in the L-cysteine product concentration by 46% in the dual-feeding, fed-batch process, reaching maximal L-cysteine concentrations of 22 g L^−1^, which are the highest reported in the literature so far [59]. The L-cysteine concentration profiles of this process before and after the strain optimization are presented in Figure 3b.

### 3.4. Purification of L-Cysteine from the Fermentation Broth

L-cysteine, due to its high solubility in water and the vulnerability of its thiol group to oxidation, presents challenges in its purification for industrial applications. Several approaches have been proposed to address this issue [62].

Most approaches follow a two-stage process, which involves converting L-cysteine into L-cystine by treating the clarified solution with an oxidizing agent. The reduced solubility and increased chemical stability of L-cystine allow for its purification through crystallization. The resulting crystals are then dissolved in water and reduced back to L-cysteine using electrolysis [71].

More recently, Wacker AG (Munich, Germany) has developed a method for the direct purification of L-cysteine from a clarified fermentation broth [62]. This approach selectively isolates L-cysteine while eliminating its derivatives, emphasizing the importance of preventing the oxidation of L-cysteine to L-cystine. The oxidation of L-cysteine’s sulfhydryl (SH) groups by oxygen, the primary oxidizing agent present in fermentation broths, is particularly favored under alkaline conditions. Additionally, thiosulfate, which serves as the preferred sulfur source for the fermentative production of L-cysteine, oxidizes L-cysteine at a pH of 5 and below. It is recommended to maintain a pH of 5–7 during fermentation to minimize any loss in yield due to the formation of L-cystine as a by-product [62].

**Figure 4:**
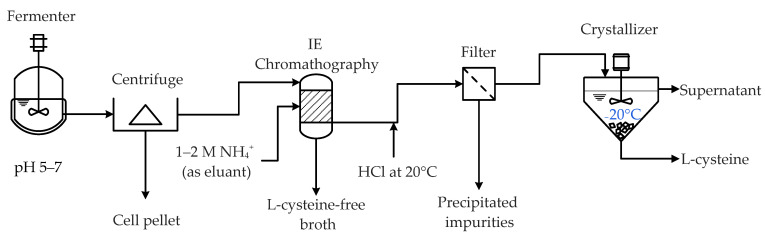
Flow diagram representation of an L-cysteine purification process according to Boehm [62]. This process relies on the L-cysteine remaining in an unoxidized state. Hence, the oxidants in the fermentation broth are removed during the anion-exchange chromatography, and the pH of the L-cysteine-rich solution must be closely monitored and adjusted throughout the purification line.

The first step in the direct purification process (Figure 4) involves ion exchange chromatography. In this step, L-cysteine, as well as L-cystine and other cations present in the clarified fermentation broth, are adsorbed onto an ion exchanger containing sulfonic and phosphonic acid groups in their protonated (H^+^) form. The absorbed compounds are subsequently eluted using ammonia solution (1–2 molar NH_4_^+^) as the eluant. The use of a gradually increasing ammonia concentration and fractioned separation of the product solution further improves the purity of L-cysteine. It is important to note that although the L-cysteine-containing solution is highly acidic, the chromatography process effectively removes all oxidizing agents, mitigating concerns about the oxidation of L-cysteine [62]. Subsequently, to further purify L-cysteine from the solution, fractioned crystallization is performed as described previously [72]. This method involves the addition of hydrochloric acid at 20 °C to facilitate the crystallization of remaining impurities. The resulting deposits are then removed through filtration, and the filtrate is cooled to −20 °C to induce the crystallization of L-cysteine.

## 4. Conclusions

In general, the most widely researched microorganism for L-cysteine bioproduction is *E. coli.* Nonetheless, other production hosts like *C. glutamicum* and *P. ananatis* have been the focus of strain optimization, with promising improvements in L-cysteine production.

The combination of three metabolic engineering strategies has been successful, irrespective of the production host: The first one is enhancing the L-cysteine biosynthesis by overexpressing the genes encoding for feedback-insensitive L-serine O-acetyltransferase (SAT) and O-acetyl-L-serine sulfhydrylase. In *E. coli*, the feedback inhibition of SAT was bypassed through the introduction of an SAT from *Arabidopsis thaliana*. *C. glutamicum* showed a high L-cysteine production with the homologous overexpression of the *cysE* and *serA* genes.

The second metabolic engineering strategy is to reduce L-cysteine degradation by the microorganisms. Therefore, the L-cysteine desulfhydrase gene *aecD* was deleted in *C. glutamicum*, which increased the production of L-cysteine. In *E. coli*, the gene products of *tnaA* and *metC* were identified to catalyze the degradation of L-cysteine, and after the deletion of these genes, the L-cysteine production was improved.

The third metabolic engineering strategy is to enhance the export of L-cysteine and weaken the import. As L-cysteine is cytotoxic, it has to be transported out of the cell. In *E. coli*, two membrane proteins called YdeD and YfiK are responsible for the export of L-cysteine. But by overexpression of *ydeD*, OAS is simultaneously secreted, which causes the L-cysteine regulon activation to stop. In contrast, the gene product of *yfiK* enables L-cysteine export without depleting the intracellular OAS concentrations. In addition, the gene product of *yeaN* is a recently discovered L-cysteine uptake protein found in *E. coli*, which may be a promising target to avoid the reconsumption of L-cysteine by the cells.

Taking into account the process engineering aspects beyond metabolic engineering of the L-cysteine biosynthesis pathway enables economic L-cysteine production by fermentation. Emphasizing energy-efficient sulfur pathways, selecting economically viable carbon sources, designing scalable fed-batch processes with individual feedings of carbon and sulfur sources, and implementing efficient purification techniques are important for the production of L-cysteine on an industrial scale. The utilization of thiosulfate instead of sulfates requires fewer reduction equivalents, and thus, enhances L-cysteine production rates. Even though the highest L-cysteine concentrations are still obtained with glucose as a carbon source, microbial L-cysteine production with glycerol has been shown. Glycerol is abundant as a by-product of the biodiesel industry, and thus, its low cost might improve the industrial-scale L-cysteine production in the future.

The different ways to enhance the bioproduction of L-cysteine show a high potential for success. A holistic approach which combines the presented methodologies may uncover synergies that may result in higher L-cysteine yields and increased productivity in the future.

## Figures and Tables

**Table 2:** ATP and NADPH demand and catalyzing enzymes for different sulfur assimilation pathways [59,63].

Pathway	Steps	Enzymes	ATP/NADPH Demand
Thiosulfate	Conversion of thiosulfate to S-sulfocysteine	CysM	-
	2. Lyase of S-sulfocysteine and sulfite	NrdH, Grx1	1 NADPH
Thiosulfate (postulated)	1. Conversion of thiosulfate to sulfite (SO_3_^−2^)	GlpE	-
	2. Reduction of sulfite to sulfide (S^−2^)	CysIJ, CysG	3 NADPH
	3. Reaction with O-acetylserine to L-cysteine	CysK	-
Sulfate	1. Activation to adenosine 5′-phosphosulfate	CysDN	1 ATP
	2. Phosphorylation to 3′-phosphoadenosine 5′-phosphosulfate	CysC	1 ATP
	3. Conversion to sulfite	CysH	1 NADPH
	4. Reduction of sulfite to sulfide	CysIJ, CysG	3 NADPH
	5. Reaction with O-acetylserine to L-cysteine	CysK	-

## Data Availability

The databases utilized in this review were Scopus and Web of Science with the keywords “cysteine biosynthesis”, “cysteine production”, and “cysteine fermentation” as search terms. The search timespan was originally set to publications between 2012 and 2023 but was extended further to cover additional metabolic approaches.
